# Telemedicine efficacy and satisfaction of patients and headache specialists in migraine management

**DOI:** 10.3389/fnmol.2023.1093287

**Published:** 2023-03-23

**Authors:** Qinlin Liu, Fang Liu, Xiran Yu, Jiali Zang, Ge Tan

**Affiliations:** Department of Neurology, The First Affiliated Hospital of Chongqing Medical University, Chongqing, China

**Keywords:** efficacy, follow-up, migraine, satisfaction, telemedicine

## Abstract

**Background:**

Migraine follow-up is difficult for outpatients, especially after the COVID-19 pandemic, we tried to identify the most appropriate telemedicine methods for migraine in terms of efficacy, safety, patient compliance, and patient and physician satisfaction.

**Methods:**

Migraine patients were screened from the Headache Center of the First Affiliated Hospital of Chongqing Medical University from September 2019 to December 2021 and randomly classified into an outpatient group and four telemedicine groups: social software, telephone, E-mail, and short message. Headache specialists followed up with the patients 3 and 6 months after their visit and asked about their satisfaction with the follow-up in each instance, as were the headache specialists.

**Results:**

A total of 147 migraine patients were included, of whom 65 completed the follow-up. After 3 and 6 months of follow-up, the proportion of patients whose monthly headache frequency decreased by over 50% in the social-software, telephone, and E-mail groups was no different from that in the outpatient group. A similar result was obtained from evaluations with the Visual Analog Scale, the Headache Impact Test and the Migraine Disability Assessment compared with baseline in social software and telephone groups. The compliance in social-software group was not worse than that in the outpatient group. The proportion of patients in the E-mail group who completed the follow-up and the proportion of patients in the telephone group who consistently took preventive medication were significantly lower than those in the outpatient group. After 6 months, the majority of patients in the outpatient, social-software, and telephone groups and headache specialists in the outpatient, social-software groups were satisfied with the follow-up, while fewer patients in the E-mail group and fewer specialists in the telephone and E-mail group showed their satisfaction.

**Conclusion:**

Compared with outpatient visits, it is safe and effective to use social software and telephone to follow up on migraine patients, and E-mail and short-message follow-up have lower feasibility. Migraine patients prefer social-software and telephone follow-up, while specialists prefer social-software follow-up.

## Introduction

Migraine is the most common type of primary headache and is a chronic disease that may follow patients throughout their lives. Approximately 1.04 billion people worldwide suffer from migraine (GBD 2016 Headache Collaborators, 2018), and the annual prevalence of migraine in adults in East Asia ranges from 4.7 to 14.3%, with a prevalence of 11 to 20% in women and 2.8 to 8.3% in men at a peak age of migraine (Takeshima et al., 2019). Migraine is now the second leading cause of disability worldwide (GBD 2016 Disease and Injury Incidence and Prevalence Collaborators, 2017), seriously affecting the health status and quality of life of many patients. Migraine has also caused great socioeconomic loss to China (Liu et al., 2013).

The current status of primary care for migraine patients is not optimistic. China is vast, but only 31 headache centers and approximately 200 headache specialist clinics have been established, which are not enough to cover all urban and rural areas (Yu et al., 2014). Due to geographical, economic, and cultural constraints, many migraine patients are unable to seek long-term care at headache specialist clinics. A total of 53.4% of Chinese patients with active headache have never sought medical care, and only 5.9% of migraine patients have been treated by advanced medical centers (Yu et al., 2014). At the same time, only 13.8% of Chinese migraine patients have correctly diagnosed (Liu et al., 2013), nearly half of whom do not use pain medication correctly, and only 2.7% of patients use migraine preventive medication in any case (Li et al., 2012). Even if migraine patients attend a headache specialist clinic, the compliance rate for chronic migraine may be only 34.5% (Yuan et al., 2020). Since the outbreak of COVID-19, strict transportation restrictions and quarantine policies in China have made cross-regional visits more difficult, and remote migraine patients’ access to care and follow-up is further limited. According to a study from the headache clinic of West China Hospital, during the pandemic, only 41.0% of patients with medication overuse headache (MOH) were able to receive regular prophylactic medications, while the majority of these patients used to receive regular prophylactic medications and approximately 50% of them experienced abrupt withdrawal (Li et al., 2021).

The duration of preventive treatment for migraine patients is usually 6 months or longer, and for the process of long-term follow-up, it is important to choose a method that is effective, safe, convenient, stable, and satisfying for both patients and physicians. With the development of the internet and electronic communication technologies, convenient access to medical care for remote areas has emerged and includes telemedicine service systems, internet hospitals, and smartphone applications. Studies from the United States and Norway have shown that remote audio and video calls for follow-up of migraine patients are feasible, effective, and safe. Compared with the usual outpatient strategy, such approaches can not only shorten the duration of involvement and improve physician efficiency but also provide high-quality specialty care for migraine patients with limited access to medical care while also lowering their expenses and delivering a high level of patient satisfaction (Müller et al., 2016, 2017a,b,c; Qubty et al., 2018; Friedman et al., 2019; Sharawat and Panda, 2021) However, due to factors such as high operating costs, confusing regulations, and the inability to include physical examinations (Weinstein et al., 2018), telemedicine has not been accepted and promoted by the general public, and there is little research on telemedicine for migraine in China.

Therefore, in this study, effectiveness, safety, patient compliance, and patient and physician satisfaction related to social-software, telephone, E-mail, and short-message approaches compared with usual outpatient care were measured for migraine patients, and the most suitable telemedicine method was selected to provide help in migraine follow-up.

## Materials and methods

### Study design

This study was a randomized, prospective observational study using data from consecutive headache patients attending the Headache Center of the First Affiliated Hospital of Chongqing Medical University from September 2019 to December 2021. All patients enrolled in this study were diagnosed with migraine for the first time. Headache patients who met the criteria for migraine in the International Classification of Headache Disorders, 3rd edition (ICHD-3) [Headache Classification Committee of the International Headache Society (IHS), 2018], were screened by 2 or more headache specialists through face-to-face interviews. The inclusion criteria were as follows: 1) age between 14 and 65; 2) at least two migraine days per month; 3) willingness to participate in the study and to complete questionnaires and follow-up. The exclusion criteria were as follows: 1) secondary headache; 2) pregnancy or lactation; 3) severe organic diseases, such as malignant tumors, severe liver and kidney damage, and myocardial infarction; 4) severe psychiatric disorders; and 5)severe visual and hearing impairment or dementia. All patients included in this study signed informed consent forms.

Next, patients were randomized to the outpatient group and four telemedicine groups: social software, telephone, E-mail, and short message. Randomization was generated by using SPSS 26.0 (IBM Corp., USA). We would first collect baseline data, and then a specified headache specialist would follow up with each patient each month after the visit in accordance with the follow-up method to which the patients had been randomly assigned, ask about their changes in headache, adjust medications in case of need, record adverse events and provide consultations for headache. The patients were asked to report the headache diaries every month and headache scales at 3 and 6 months after the visit. At the end of the 6-month follow-up, we asked the patients and headache specialists if they were satisfied with the follow-up methods and why.

The follow-up patients of the outpatient group were required to come to the headache clinic once a month after the first visit, and headache specialists would conduct face-to-face, one-on-one consultations with the patients. In the social software group, we used WeChat, the most widely used social platform in China, to follow up with every patient. The headache specialist regularly communicated with the patient on the WeChat platform every month after the first visit, by sending text and voice messages or making voice and video calls. In the telephone group, the headache specialists would call their patients once a month, record the headache changes through voice consultation, and give corresponding treatment suggestions. Similarly, the headache specialists in the E-mail and short message groups sent emails and short messages to patients every month, reminding them to send the headache diaries or headache scale to the mailbox and mobile phone number of the regular follow-up doctor. Corresponding treatment advice was also sent to headache patients *via* E-mail and short messages.

### Measures

Baseline data included sex, age, age of onset, duration of the attack, headache days, migraine days, visual analog scale (VAS), Headache Impact Test (HIT-6), Migraine Disability Assessment (MIDAS), preventive and analgesic treatment, etc.

We used the VAS to measure headache severity, with a score of 0 indicating no headache and 10 indicating a very severe headache. The HIT-6 was used to measure the burden of headache in the patient’s daily life, with higher scores indicating a higher degree of impact on daily life (Nachit-Ouinekh et al., 2005). The MIDAS provided an assessment of the degree of disability due to migraine, taking into account days of work and household chores affected by headaches in the past 3 months (Stewart et al., 2001). Patients were asked to record headache diaries for each attack, reporting onset time, headache sites, VAS, accompanying symptoms, use of analgesic medication, and duration of the attack. The headache diaries would help specialists evaluate the changes in headache once a month so that the treatment plan could be adjusted if necessary.

The primary efficacy endpoint of the study was a ≥ 50% decrease in monthly migraine days, and secondary efficacy endpoints included a ≥ 50% decrease in monthly headache days, headache days, migraine days, VAS, HIT-6, and MIDAS scores. We assessed the safety of the follow-up method by determining the proportion of patients reporting adverse events in each group during the 6 months. Compliance was evaluated from the percentage of patients completing follow-up in each group at 3 and 6 months and from the percentage of patients who consistently took preventive medication. Satisfaction was assessed by determining the percentage of patients and headache specialists in each group who were satisfied with the follow-up method.

### Statistical methods

The differences in age, age of onset, duration of the attack, frequency, VAS, HIT-6, and MIDAS in all groups were statistically analyzed using the one-way ANOVA. The proportions of the female sex, chronic migraine (CM), and MOH were analyzed using Pearson’s chi-square test, while we chose Fisher’s exact test if expectation<5. We used the one-way ANOVA with multiple comparisons to analyze differences in headache days, migraine days, VAS, HIT-6, and MIDAS within and with different groups at follow-up at 3 and 6 months. We used Pearson’s chi-square test to analyze the proportions of patients with a 50% decrease in migraine and headache days, patients reporting adverse events, patients consistently taking preventive medicines, patients completing the 6-month follow-up, patients satisfied with the follow-up methods and headache specialists satisfied with the follow-up methods in different groups at 3 and 6 months. We chose Fisher’s exact test if expectation <5. In addition, we have performed a power analysis on the statistics of telemedicine efficacy, safety, compliance, and satisfaction. Statistical analysis was performed for the outpatient group and the sum of all telemedicine groups for indicators with lower Power values. *p* < 0.05 was considered statistically significant with a 95% confidence interval. All data were analyzed using SPSS 26.0 (IBM Corp., USA), GraphPad Prism 8, and GPower 3.1.

## Results

### Participation

**Figure 1:**
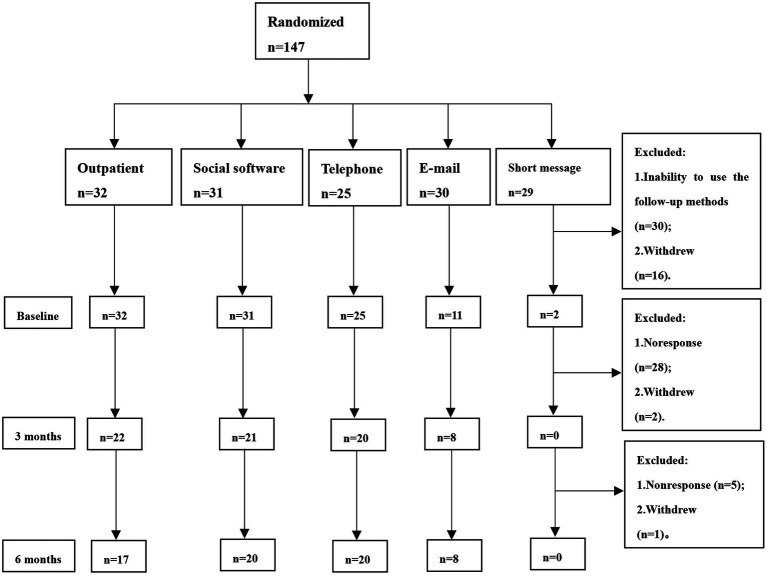
Study flow.

A total of 147 migraine patients were included in this study. Before the collection of baseline data, 30 patients were excluded because of their inability to use the follow-up method that was randomly assigned. Another 16 patients dropped out of the study, and a total of 101 patients completed baseline data collection. At the 3-month follow-up, 30 patients were excluded, 28 of whom were not responsive and 2 of whom withdrew from the study. At the 6-month follow-up, 6 patients were excluded, 5 of whom were not responsive and 1 of whom withdrew from the study. Finally, a total of 65 patients completed follow-up (Figure 1). Because no patients in the short-message group completed the 6-month follow-up, this group’s data were not included in statistics and analysis.

### Baseline demographics

The baseline data are shown in Table 1. There were no significant differences in the different groups. However, the E-mail group revealed a lower age of onset (29.8 ± 9.6 vs. 20.4 ± 7.8, *p* = 0.206), headache days (14.9 ± 9.3 vs. 6.4 ± 5.7, *p* = 0.194), migraine days (10.9 ± 8.1 vs. 4.3 ± 3.3, *p* = 0.190) and proportion of CM (11, 64.7% vs. 1, 12.5%, *p* = 0.098).

**Table 1:** Baseline demographics in each group.

	Outpatient	Social software	Telephone	E-mail	*P*
Female, *n*(%)	14(82.4)	16(80.0)	15(75.0)	6(75.0)	0.974
Age, mean ± SD	42.3 ± 12.8	41.2 ± 12.3	41.7 ± 14.9	33.0 ± 14.6	0.494
Age of onset, mean ± SD	29.8 ± 9.6	30.8 ± 11.3	27.1 ± 12.1	**20.4 ± 7.8**	0.206
Duration, h	16.8 ± 6.8	18.9 ± 12.0	17.3 ± 8.6	15.0 ± 12.7	0.895
VAS, mean ± SD	6.8 ± 1.0	6.7 ± 1.0	6.7 ± 1.3	6.9 ± 1.3	0.958
HIT-6, mean ± SD	65.9 ± 18.7	62.8 ± 8.0	63.3 ± 7.7	63.4 ± 4.3	0.860
MIDAS, mean ± SD	81.1 ± 79.6	80.5 ± 64.6	84.5 ± 73.7	31.8 ± 33.3	0.324
Headache days, mean ± SD	14.9 ± 9.3	14.0 ± 10.6	15.1 ± 10.6	**6.4 ± 5.7**	0.194
Migraine days, mean ± SD	10.9 ± 8.1	10.3 ± 7.8	11.4 ± 8.9	**4.3 ± 3.3**	0.190
CM, *n*(%)	11(64.7)	9(45.0)	11(55.0)	**1(12.5)**	0.098
MOH, *n*(%)	2(11.8)	4(20.0)	5(25.0)	0	0.477

CM, chronic migraine; HIT-6, Headache Impact Test-6; MIDAS, Migraine Disability Assessment; MOH, medication-overuse headache; SD, standard deviation; VAS, Visual Analogue Scale. The bold content indicates that the values or proportions are different from those within or between groups, with or without statistical significance.

### Efficacy

**Figure 2:**
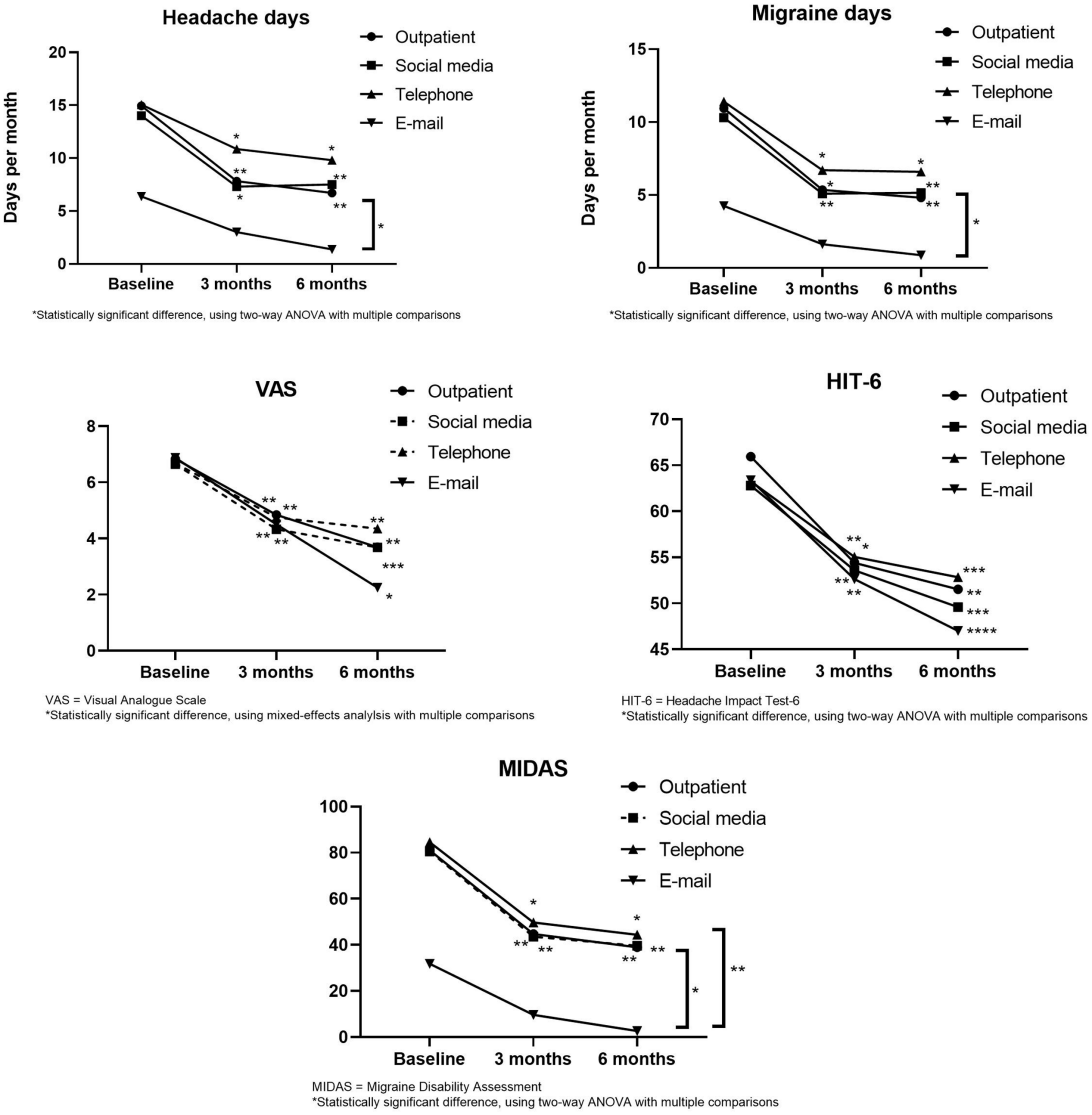
Headache days, migraine days, VAS, HIT-6 and MIDAS during 6  months.

Figure 2 shows that headache days, migraine days, VAS, HIT-6, and MIDAS in each group decreased with the extension of follow-up time. After 6 months of follow-up, VAS and HIT-6 in all follow-up groups were significantly different from the baseline. In the email group, we saw a trend towards reductions in headache days, migraine days, and MIDAS, but not as statistically significant as in the other groups.

In comparing the differences between groups, the headache days, migraine days, and MIDAS in the E-mail group at 6 months of follow-up were significantly lower than those in the other groups. The headache days and migraine days in the telephone group were slightly higher than that in the outpatient group at 3 months and 6 months, but there was no statistical significance (Figure 2).

There were no significant differences between the groups in the proportion of patients with a ≥50% reduction in headache days and a ≥50% reduction in migraine days at 3 and 6 months of follow-up. Due to low statistical power, we compared the outpatient group with all telemedicine groups and still found no statistical difference (Table 2).

**Table 2:** Proportion of monthly headache days and migraine days decreased by ≥50% after follow-up.

	3 months		6 months	
	Migraine days decreased by ≥50%, *n*(%)	Headache days decreased by ≥50%, *n*(%)	Migraine days decreased by ≥50%, *n*(%)	Headache days decreased by ≥50%, *n*(%)
Outpatient	11(64.7)	9(52.9)	13(76.5)	11(64.7)
Social software	12(60.0)	11(55.0)	14(70.0)	13(65.0)
Telephone	12(60.0)	9(45.0)	12(60.0)	11(55.0)
E-mail	7(87.5)	5(62.5)	8(100.0)	8(100.0)
*P*	0.548	0.879	0.186	0.150
Power	0.678	0.295	0.987	0.969
Telemedicine	31(64.6)	25(52.1)	34(70.8)	32(66.7)
*P*	0.993	0.951	0.760	0.883

### Safety

During the 6-month follow-up (Table 3), there were 3 cases (17.6%) reporting mild to moderate adverse events in the outpatient group, including 2 cases with stiffness and weakness of the injected muscle after botulinumtoxin A injection, all relieved after the 3-month follow-up; the other case involved sleepiness after taking pregabalin, and the sleepiness was relieved after the patient reduced the dose of pregabalin on her own. There were 4 cases (20.0%) reporting mild to moderate adverse events in the social-software group, of which 2 involved flunarizine intake-related fatigue and obesity that was relieved after flunarizine was stopped and topiramate started; 1 patient had numbness in the fingers after taking topiramate, and the numbness was relieved after approximately 1 week without attention; and 1 patient had stiffness and weakness of the injected muscle after botulinumtoxin A injection, which was relieved after the 3-month follow-up. In the telephone group, 1 patient (5.0%) reported finger numbness after taking topiramate, which was relieved after the patient decided on her own to stop taking the drug. In the E-mail group, 1 patient (1.3%) reported sleepiness after taking flunarizine but obtained relief by taking it earlier. The proportion of adverse events in each group was not significantly different from that in the outpatient group. No serious adverse events occurred during the entire follow-up period.

**Table 3:** Adverse events in each group during the 6  months.

	Adverse events, *n*(%)	*P*	Power
Outpatient	3(17.6)	0.545	1.000
Social software	4(20.0)		
Telephone	1(5.0)		
E-mail	1(1.3)		

### Compliance

After the 3-month follow-up, the numbers of patients who completed the follow-up in the outpatient, social-software, telephone, and E-mail groups were 22 (68.8%), 21 (67.7%), 20 (80.0%), and 8 (26.7%), respectively. After 6 months, the numbers in each group decreased to 17 (53.1%), 20 (64.5%), 20 (80.0%), and 8 (26.7%), respectively. Among them, the proportion of follow-up patients in the E-mail group at 3 months (*p* < 0.001) and 6 months (*p* = 0.001) were significantly lower than those in the other groups (Table 4).

**Table 4:** Compliance at 3 and 6 months in each group.

		Completed the follow-up, *n*(%)	*P*	Power	Consistently took preventive medication, *n*(%)	*P*	Power
3 months	Outpatient	22(68.8)	<0.001*	1.000	17(77.3)	0.017*	1.000
	Social software	21(67.7)			16(76.2)		
	Telephone	20(80.0)			**9(45.0)**		
	E-mail	**8(26.7)**			8(100.0)		
6 months	Outpatient	17(53.1)	0.001*	0.969	13(76.5)	0.003*	1.000
	Social software	20(64.5)			15(75.0)		
	Telephone	20(80.0)			**7(35.0)**		
	E-mail	**8(26.7)**			8(100.0)		

*Statistically significant difference, using Pearson Chi-square Tests or Fisher’s Exact Tests as appropriate. The bold content indicates that the values or proportions are different from those within or between groups, with or without statistical significance.

By 3 months, 17(77.3%), 16(76.2%), 9(45.0%), and 8(100.0%) of the follow-up patients in the outpatient, social software, telephone, and E-mail group were consistently taking preventive medication. After 6 months, the number decreased to 13 (76.5%), 15 (75.0%),7 (35.0%), and 8 (100.0%), respectively. Among them, the proportion of patients in the telephone group who consistently took preventive medicines at the 3-month (*p* = 0.017) and 6-month (*p* = 0.003) follow-ups was significantly lower than the other groups (Table 4).

### Satisfaction

After 6 months of follow-up, most of the patients and headache specialists were satisfied with the follow-up method. In the outpatient group, 17 (100.0%) of the migraine patients and headache specialists were satisfied with the follow-up method. The number of migraine patients and headache specialists satisfied with the follow-up method was 19 (95.0%) and 20 (100.0%) in the social-software group and 19 (95.0%) and 15 (75.0%) in the telephone group, respectively. In the E-mail group, 7 (87.5%) migraine patients and 7 (87.5%) headache specialists were satisfied with the follow-up method. It is worth mentioning that in the telephone group, the satisfaction on the part of headache specialists was much lower than that of patients and the other groups. Patients who were dissatisfied said that telephone follow-up interfered with their normal life, and headache specialists complained about low communication efficiency, involvement during both rest and work time and difficulty in carrying out subsequent work. We found that fewer patients and headache specialists were satisfied with the follow-up method in the E-mail group than in the outpatient group, but the difference was not statistically significant. The reasons given by patients who were dissatisfied with the follow-up method were that E-mail contact was not convenient enough, and there were so many spam E-mails that it was easy to miss the specialist’s messages. The reason given by headache specialists was that organizing E-mail was not clear enough (Table 5).

**Table 5:** Satisfaction of patients and specialists after 6 months in each group.

	Patients satisfied, *n*(%)	Specialists satisfied, *n*(%)
Outpatient	17(100.0)	17(100.0)
Social software	19(95.0)	20(100.0)
Telephone	19(95.0)	**15(75.0)**
E-mail	7(87.5)	7(87.5)
*P*	0.522	0.012*
Power	1.000	1.000

*Statistically significant difference, using Pearson Chi-square Tests or Fisher’s Exact Tests as appropriate. The bold content indicates that the values or proportions are different from those within or between groups, with or without statistical significance.

## Discussion

At present, there are many studies on various ways to follow up with patients using telephone, video, internet platforms, smartphone apps, etc., but there is no study of telemedicine in migraine treatment in China. Our study is the first to investigate the efficacy, safety, compliance, and satisfaction of follow-up in relationship to migraine, and we hope to identify the most appropriate telemedicine approach for the treatment of migraine.

The majority of patients in the short-message group dropped out of the study before completing baseline data collection, and no patients were followed up after 3 months (Figure 1). We think that this may be related to the following reasons: (1) Communication is inconvenient. Most short messages can only be sent by text, which requires considerable time typing and reduces communication efficiency. It is also difficult for many patients to report their symptoms completely and send the scales by text message; (2) It is inconvenient to organize data. Patients receive various short messages every day, such as notifications, announcements, and advertisements, and they may miss the messages from headache specialists. (3) Fees. It may cost extra to send short messages. If patients send messages with pictures, audio, or video, the fee on their phone bills will be much higher. In contrast, patients need to pay only a fixed monthly network fee for social software and E-mail.

We did not require patients to be able to use all follow-up methods since this would inevitably exclude a significant proportion of older, undereducated patients living in remote areas who might not use electronic devices and might even be illiterate. This study discusses how to effectively follow up with patients who are unable to attend outpatient clinics due to geographical, economic, and other factors. Compliance in remote areas is very important to us, so we cannot exclude such patients. At the same time, low compliance reflects the limitations and difficulties of follow-up methods. We believe that for telemedicine, it is critical to choose a method that facilitates doctor-patient communication. Therefore, low compliance means the low feasibility of the telemedicine method to a certain extent and serves as a reference for clinicians to choose a more reliable telemedicine method.

The results showed that age and age of onset in the E-mail group were lower than in the other groups (Table 1). We think that this result may be because the use of E-mail places special requirements on patients. A survey from Australia on E-mail communication with patients indicated that some patients with limited literacy or no access to the internet may be disadvantaged in accessing health care by E-mail (Clark et al., 2021). Therefore, in our study, some patients, limited by economic conditions and education levels, may have been unable to use E-mail. These patients were randomly assigned to the E-mail group and were excluded after the baseline data collection. In contrast, the baseline data in the social-software group and the telephone group were not significantly different from those in the outpatient group. This suggests that the popularity of smartphones is relatively high, and using social software and telephone to follow up on migraine is feasible and convenient.

The results in Table 1 show that the headache days, migraine days, proportion of CM, and MIDAS scores in the E-mail group were lower than the other groups. It illustrates that patients with more frequent headache attacks are less likely to use E-mail to communicate with their specialists during follow-up than to visit outpatient clinics or to use social software or phone calls. This is not only related to the inconvenience and unpopularity of E-mail but also the different preferences of patients with low headache frequency, but the reasons for this preference are still unclear. Current research shows that most CM evolves from episodic migraine (EM) with an annual progression rate of approximately 3%. The chronicity of migraine is very complicated. CM and EM patients have different characteristics in terms of genetic, physiological, and psychosocial factors. However, the key structures and networks involved in the phenomenon of chronicity are not fully understood, and further research is needed (Buse et al., 2012; May and Schulte, 2016).

Table 2 shows that after 6 months of follow-up, no significant difference was found between telemedicine groups and outpatients in the efficacy of migraine treatment. This also shows that the use of social software, telephone, and E-mail to follow up with migraine patients is effective in headache relief, and the results are similar to those of studies from Norway and the United States. Müller et al. from Norway divided CM patients into two groups: a video telemedicine group and an outpatient group. These patients were diagnosed and treated by the two methods and followed up for up to 1 year. There were no statistically significant differences between the two groups in VAS and HIT-6 scores or the decrease in VAS and HIT-6 scores compared with baseline, and there was also no significant difference in the rate of misdiagnosis between the two groups (Müller et al., 2016, 2017c). Friedman et al. in the United States separated migraine patients into a video telemedicine group and an outpatient group. Their results showed that the MIDAS scores, monthly headache days, and average headache severity in the telemedicine group were significantly different from those in the outpatient group (Müller et al., 2017c).

At 3 and 6 months of follow-up, the headache and migraine days of the patients in the telephone group were slightly higher than that of patients in the outpatient group, but VAS, HIT-6, and MIDAS scores as well as the proportion of patients whose monthly headache and migraine days decreased by ≥50% were not significantly different from those of outpatients (Figure 2). We think that after receiving migraine education during follow-up, patients began to learn migraine self-management, including keeping headache diaries, taking analgesic medication correctly, adjusting lifestyle habits, and maintaining a healthy psychological state. As a result, the frequency, severity, and duration of headaches decreased, and the impact of headaches on their daily lives was reduced, which allowed them to avoid the disabled condition due to headaches as much as possible. Meanwhile, headache specialists can better understand the changes in headache through follow-up to provide more accurate and effective advice. The results of the study by David B. Matchar et al. are similar to ours. They randomly assigned headache patients to a control group and an intervention group, and those in the intervention group received a comprehensive headache management program, including educational sessions, visits for evaluation, and follow-up visits. After the 6-month follow-up, the MIDAS scores in the intervention group improved more than those in the control group, as did the overall quality of life and satisfaction (Matchar et al., 2008). Therefore, in addition to medication, effective headache management can reduce disability in migraine patients.

In our study, most adverse events were resolved after contacting the headache specialist, but there were still 2 patients who did not contact us and who adjusted their medication on their own, including 1 patient in the outpatient group and another in the telephone group (Table 3). We found that compared with outpatient visits, the other telemedicine methods made it easier for patients to contact headache specialists, and the patients could get advice very quickly. Although there was no serious adverse event during the study, we still recommend that patients call the emergency number or go to the hospital in the event of a serious adverse event as obtaining immediate assistance may be limited when they make an appointment in the headache center or contact a headache specialist.

Table 4 shows that the follow-up rate in social-software and telephone follow-up methods was not lower than that observed for outpatient visits, and the follow-up rate was highest with telephone. This is because telephone contact is one of the most convenient ways of communication, while with social-software, e-mail, and short-message approaches, patients may miss the communications, fail to receive notifications, and not be bothered to reply. Among all follow-up methods, E-mail had the lowest follow-up rate, which is easy to understand. In addition to the above reasons, previous studies have proven that being able to use E-mail is affected by economics and education (Clark et al., 2021), and some patients may withdraw from the study due to their inability to use E-mail.

The results revealed that the proportion of patients consistently taking medication in the telephone group was significantly lower than that in the other groups (Table 4). We think this is because there is immediacy in making and receiving phone calls. The migraine specialists may be busy with other affairs while making the call, and the hasty communication will cause specialists to inadequately understand the headache changes and be unable to give effective advice. It is easy for patients to misunderstand medication advice in such hurried communications. All of these things can lead to low effectiveness and compliance. This was also reflected in satisfaction; the satisfaction of specialists in the telephone group was not only lower than that of patients in the same group but also lower than that of specialists in other follow-up groups (Table 5). We asked these specialists why they said that compared with the outpatient approach, telephone follow-up was sometimes problematic in terms of communication efficiency; telephone communication occupied not only their working time but also their resting time, leading to difficulty in carrying out subsequent work. Some specialists were not satisfied with and even contradicted the value of follow-up by telephone.

In addition, although some patients and specialists were dissatisfied with the E-mail follow-up method due to the inconvenience of sending, receiving, and sorting E-mail, there was no significant difference.

Our study has some limitations. Even though we repeatedly emphasized to patients that their satisfaction rating should not relate to efficacy but, rather, the method only, the reasons for patients dropping out of the study were still poor efficacy, adverse events, or other unclear factors, which we could not collect due to nonresponse. Thus, we may have overestimated the satisfaction of migraine patients and the proportion of patients who consistently took medication, and we may have underestimated the adverse events. Moreover, since the patients included in the study came from a single center, the sample size was relatively small. Thus, we conducted a power analysis on the comparison of the differences among the groups to improve the effectiveness of the statistical analysis. For indicators with low Power values, we performed statistical analysis between the outpatient group and the sum of all telemedicine groups. Furthermore, patients were randomly assigned to different follow-up methods in this study, and we hypothesized that if patients could voluntarily choose the follow-up method, the effectiveness, compliance, and satisfaction of migraine management may be improved. Of course, more controlled trials are needed to verify the influence of that.

## Conclusion

Compared with outpatient visits, it is safe and effective to use social software, telephone, and E-mail to follow up with migraine patients, but E-mail and short-message follow-up are less feasible. Most migraine patients and specialists are satisfied with social-software, telephone, or E-mail follow-up; migraine patients prefer social-software and telephone follow-up, while specialists prefer social-software follow-up.

## Data availability statement

The raw data supporting the conclusions of this article will be made available by the authors, without undue reservation.

## Ethics statement

This study received ethical approval from the Ethics Committee of the First Affiliated Hospital of Chongqing Medical University (20190716). The patients/participants provided their written informed consent to participate in this study.

## Author contributions

GT conceived and oversaw the study. QL, XY, and JZ completed the data collection. QL performed data analysis. QL, FL, and GT wrote and revied the manuscript. All authors read and approved the final manuscript.

## Funding

This study was supported by the Chongqing Medical Scientific Research Project of the Chongqing Health Commission and Science and Technology Bureau (2020MSXM096).

## Conflict of interest

The authors declare that the research was conducted in the absence of any commercial or financial relationships that could be construed as a potential conflict of interest.

## Publisher’s note

All claims expressed in this article are solely those of the authors and do not necessarily represent those of their affiliated organizations, or those of the publisher, the editors and the reviewers. Any product that may be evaluated in this article, or claim that may be made by its manufacturer, is not guaranteed or endorsed by the publisher.

## Abbreviations

ICHD-3: the International Classification of Headache Disorders, 3rd edition
VAS: visual analogue scale
HIT-6: Headache Impact Test
MIDAS: Migraine Disability Assessment
CM: Chronic migraine
MOH: medication overuse headache
